# Anti-Lan Antibodies: A Rare Etiology of Severe Blood Transfusion Reaction

**DOI:** 10.7759/cureus.10832

**Published:** 2020-10-06

**Authors:** Purva Sharma, Sukesh Manthri, Emily Patterson, Bahaaeldin Youssef, Kanishka Chakraborty

**Affiliations:** 1 Medical Oncology, East Tennessee State University, Johnson City, USA; 2 Oncology, East Tennessee State University, Johnson City, USA; 3 Pathology, East Tennessee State University, Johnson City, USA

**Keywords:** anti-lan antibodies, transfusion reaction, clinical immunology, blood group

## Abstract

Lan is a high prevalence red blood cell antigen present in the majority of the populations that belong to the Lan (Langereis) blood group system. Anti-Lan antibody is an immunoglobulin G (IgG) antibody that is known to cause delayed hemolytic transfusion reactions in adults as well as hemolytic disease in fetuses and newborns, however with variable clinical significance ranging from mild to severe. We present a 58-year-old woman with diffuse abdominal pain and a large gastric ulcer causing gastric outlet obstruction. She underwent antrectomy and Billroth I reconstruction surgery without complications. The patient’s hemoglobin upon presentation was 10g/dL and dropped acutely post-operatively to 6.4 g/dL requiring blood transfusion. The patient developed acute respiratory distress within minutes of starting a packed red blood cell (pRBC) transfusion, requiring discontinuation. Laboratory testing demonstrated pan-reactivity with additional reference testing demonstrating an anti-Lan antibody. The rarity of Lan negative pRBC units is a challenge in managing such patients requiring blood transfusions. Autologous blood donation or donation by a compatible family member is another option to consider in these rare cases.

## Introduction

Lan is a high prevalence red blood cell antigen that belongs to the Lan (Langereis) blood group system and is present in >99% of all populations [1]. Anti-Lan antibody is an immunoglobulin G (IgG) antibody that is known to cause delayed hemolytic transfusion reactions in adults as well as hemolytic disease in fetuses and newborns, however with variable clinical significance. We present an intriguing case of a 58-year-old woman with a history of several significant blood transfusion reactions in the past at an outside hospital (unknown reaction etiology), who was found to have Lan negative phenotype with anti-Lan antibody.

## Case presentation

A 58-year-old Caucasian woman with a history of prior transfusion reactions at an outside institution and known gastric ulcer refractory to medical therapy presented with diffuse abdominal pain associated with persistent nausea and vomiting. She reported decreased oral intake and weight loss for the last couple of months. Her medical history was significant for normocytic anemia requiring iron infusions. On laboratory evaluation, her hemoglobin was 10 g/dL, with normal white blood cell and platelet counts. Computerized tomography of the abdomen/pelvis with contrast was unremarkable. Upper gastrointestinal (GI) endoscopy revealed partial gastric outlet obstruction secondary to a large gastric ulcer. She was status post truncal vagotomy, antrectomy, and Billroth I reconstruction with no immediate complications. However, her hemoglobin acutely dropped from 10g/dL to 6.4g/dL post-operatively, with no obvious source of bleeding. Shortly into a unit of least incompatible phenotypically matched pRBCs she experienced acute respiratory distress, requiring 6L/min supplemental oxygen. The transfusion was stopped and a transfusion reaction workup was initiated.

The patient’s blood group was previously identified as type O, Rh type positive. Initial serologic workup performed at an outside institution in the year 2000 revealed a high titer low avidity antibody (HTLA) and anti-E phenotype testing demonstrated the patient to lack Fy(b) and S.

Workup at our institution showed positive screen cells and pan-reactivity (2+ strength) on the antigen profile panel with a negative auto control. Following the above-described transfusion reaction, additional testing was performed. No visible hemolysis was present. Direct Coomb’s testing was negative. Two days later, testing showed positive polyspecific direct antiglobulin test (weak), positive (+1) Anti-C3b, and positive (+1) Anti-C3d. Anti-IgG was negative. Given the unusual nature of this reaction, send-out testing at a reference lab was performed with an eluate prepared from the patient’s red cells demonstrating anti-Lan specificity. Anti-Lan demonstrated reactivity by indirect antiglobulin test (IAT), polyethylene glycol IAT (PEG IAT), and Ficin IAT. The patient’s genomic DNA was isolated and red cell genotyping was performed to determine the predicted red cell phenotype for selected red cell antigens in the Rh, Kell, Duffy, Kidd, MNS, Lutheran, Dombrock, Colton, Cromer, Yt, Diego, and Vel blood group systems and to investigate the presence of a hybrid D-CE-D allele. No variant or null alleles were detected. The patient’s predicted RH genotype is Ce/Ce and the predicted phenotype E was consistent with anti-E alloimmunization.

**Figure 1:**
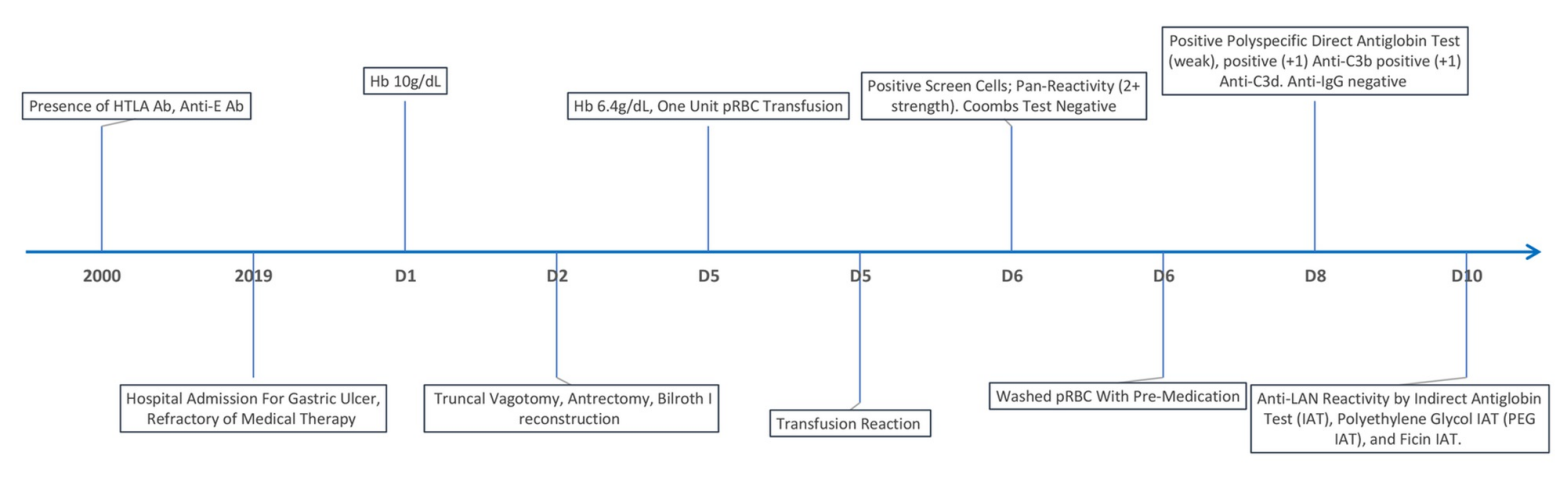
Timeline of the patient's clinical course D - day; HTLA - high titer low avidity; Hb - hemoglobin; pRBC - packed red blood cell; IgG - immunoglobulin G; Ab - antibodies

As the patient was clinically symptomatic, she was transfused three units of washed pRBCs after premedication with methylprednisone and acetaminophen since the availability of Lan negative units of blood is extremely rare. She tolerated the transfusions well, with no signs or symptoms of subsequent transfusion reactions. Figure 1 depicts the patient's clinical course.

The patient tolerated the washed units of pRBCs well, without any further events of transfusion reactions. She was discharged to a rehabilitation facility once her hemoglobin stabilized. The patient’s primary care physician and hematologist were notified of the work-up and hematological diagnosis. The patient has two siblings, and it was recommended to test her siblings for possible Lan-negative donors if required in the future.

## Discussion

The anti-Lan antibody was first described in 1962 as an antibody to high prevalence red blood cell antigen responsible for severe and acute hemolytic transfusion reaction [2]. Lan is a high prevalence antigen (>99.9%), and Lan antigen negativity is considered a rare blood type worldwide [1]. Prevalence of the Lan negative type was estimated to be approximately one in 20,000 in Caucasians, one in 50,000 in Japanese, and one in 1,500 in black people from South Africa [3-6]. The Lan negative phenotype is inherited as a recessive character. 

The adenosine triphosphate (ATP)-binding cassette (ABC) molecule, subfamily B, member 6, also known as ABCB6 protein, is the carrier of the Lan blood group antigen [7, 8]. The ABCB6 gene (chromosome 2q36, 19 exons) encodes the ABCB6 polypeptide known as a porphyrin transporter [9]. Patients with Lan negative phenotype reported to date were seemingly healthy, experiencing no clinical symptoms of porphyria or abnormal complete blood count [10]. As a result, ABCB6 is known not to be a protein essential for life in humans, and it does not appear to be fully required for erythropoiesis.

Anti-Lan antibody may be stimulated by pRBCs transfusion or pregnancy. No naturally occurring anti-Lan antibody has been reported to date [11]. Our patient was nulliparous, and presumably, she developed the antibody through exposure to the Lan antigen in one of her known prior blood transfusions. Lan alloantibodies are mostly a mix of immunoglobulin (Ig) G1 and IgG3, but IgG1 or IgG3 alone has also been described, as well as IgG2 and IgG4 components. Anti-Lan may fix complement. Of note, our patient’s initial direct Coombs test was negative. A follow-up Coombs test, including complement, was positive. The presence of the positive Coombs test two days after transfusion with further work-up showing anti-LAN specificity is compatible with a delayed serologic transfusion reaction. Clinically, hemolysis markers, including lactate dehydrogenase (LDH) and haptoglobin, were unremarkable, and she did not show features of hemolysis during the follow-up period after the reaction.

Anti-Lan antibody can have variable clinical significance. It may cause hemolytic transfusion reactions, which may be mild or severe [12]. It has also been shown to cause hemolytic disease of the newborn [13]. This patient’s transfusion reactions were variable in terms of severity ranging from no reaction to severe reactions following red cell transfusions. Following the completion of transfusion work-up documenting anti-Lan antibody, and due to the unavailability of Lan negative pRBCs, for subsequent transfusions, our patient received washed packed red blood cells after premedication with methylprednisone. She tolerated several washed pRBC transfusions without any transfusion reactions. One possible reason could be that she had low titers of anti-Lan antibody and hence did not develop any significant reaction. 

The etiology for this patient’s transfusions reactions is not entirely clear. The patient’s symptoms during her severe transfusion reactions were predominately respiratory in nature with one reaction showing a mild fever of 99 F rather than the classically reported hemolytic reaction. Transfusion-related acute lung injury (TRALI) was clinically excluded. Her unusual transfusion reaction history is further complicated by significant underlying comorbidities, including chronic obstructive pulmonary disease (COPD) and congestive heart failure (CHF). It is curious that no transfusion reactions were reported in this patient when she received washed red cell products. Washed products are typically utilized for the prevention of severe allergic transfusion reactions or in cases of repeated febrile non-hemolytic transfusion reactions.

Overall, the etiology for this patient’s unusual transfusion reactions is complicated both by the lack of reported transfusion reactions for the rare anti-LAN antibody and by confounding factors in the patient’s known underlying comorbidities. The development of a positive Coombs test two days after transfusion with elution showing anti-LAN specificity confirms at least a laboratory serologic link between anti-LAN and these reactions. Although hemolytic anti-LAN reactions are documented, this patient’s unusual reactions raise the possibility that non-hemolytic transfusion reactions with respiratory distress related to anti-LAN may also occur. 

The patient has two living siblings. Results of the patient’s workup were discussed with the patient’s primary care physician and appropriate testing of the patient’s siblings was recommended to evaluate for possible future Lan negative donors. Results of that are not available at the time of writing this paper. 

## Conclusions

Lan is a high prevalence antigen (>99.9%) and Lan negative is considered a rare blood type world-wide. The clinical significance of the presence of anti-Lan antibody is variable, with mild or severe hemolytic reaction to no reaction. Despite challenging conditions caused by scarcity of Lan negative donors worldwide, Lan negative RBC units should ideally be selected for the transfusion of Lan negative patients with anti-Lan antibody, especially those with a high-titer antibody. Transfusion with Lan positive units might be required, but close monitoring for hemolysis is necessary in such instances. Autologous blood donation or donation by a compatible family member is another option.
